# Quantitative anatomy of the primary ossification center of the radial shaft in human fetuses

**DOI:** 10.1007/s00276-019-02247-2

**Published:** 2019-05-02

**Authors:** Marcin Wiśniewski, Mariusz Baumgart, Magdalena Grzonkowska, Zygmunt Siedlecki, Maciej Piec, Michał Szpinda, Katarzyna Pawlak-Osińska

**Affiliations:** 1 Department of Normal Anatomy, The Ludwik Rydygier Collegium Medicum in Bydgoszcz, Łukasiewicza 1 Street, Bydgoszcz, 85-821 Poland; 2 Department of Neurosurgery, Neurotraumatology and Pediatric Neurosurgery, The Ludwik Rydygier Collegium Medicum in Bydgoszcz, The Nicolaus Copernicus University in Toruń, Toruń, Poland; 3 Department of Positron Emission Tomography and Molecular Imaging, The Ludwik Rydygier Collegium Medicum in Bydgoszcz, The Nicolaus Copernicus University in Toruń, Toruń, Poland; 4 Department of Otolaryngology and Oncology, The Ludwik Rydygier Collegium Medicum in Bydgoszcz, The Nicolaus Copernicus University in Toruń, Toruń, Poland

**Keywords:** Radius, Bone development, Osteogenesis, Fetal development

## Abstract

**Purpose:**

The medical literature still lacks studies on the size of the radial shaft primary ossification center, thus preventing us from potentially relevant data in diagnosing skeletal dysplasias, i.e., TAR syndrome, VATER syndrome, Holt–Oram syndrome, Fanconi anemia and Edwards syndrome, frequently characterized by disrupted or retarded fetal growth.

**Materials and methods:**

The size of the radial shaft primary ossification center in 47 (25 males and 22 females) spontaneously aborted human fetuses aged 17–30 weeks was studied by means of CT, digital image analysis and statistics.

**Results:**

With neither sex nor laterality differences, the best-fit growth dynamics for the radial shaft primary ossification center was modeled by the following functions: *y* = − 10.988 + 1.565 × age ± 0.018 for its length, *y* = − 2.969 + 0.266 × age ± 0.01 for its proximal transverse diameter, *y* = − 0.702 + 0.109 × age ± 0.018 for its middle transverse diameter, *y* = − 2.358 + 0.203 × age ± 0.018 for its distal transverse diameter, y = –189.992 + 11.788 × (age)^2^ ± 0.018 for its projection surface area, and *y* = − 798.174 + 51.152 × age ± 0.018 for its volume.

**Conclusions:**

The morphometric characteristics of the radial shaft primary ossification center show neither sex nor bilateral differences. The radial shaft primary ossification center grows proportionately in length, transverse dimensions and volume, and quadratically in its projection surface area. The obtained numerical findings of the radial shaft ossification center are considered age-specific reference of relevance in both the estimation of fetal ages and the diagnostic process of congenital defects.

## Introduction

During weeks 6 and 7 of gestation, the cartilaginous skeleton of the upper limb is designed from the mesenchyme. At that time, the only exception to this refers to distal phalanges that are still mesenchymal. Except for the clavicle, all the remaining bones of the upper limb ossify from cartilage due to endochondral ossification [1]. The ossification process of the upper limb commences at the end of week 6 of gestation with the formation of the primary ossification center in the middle part of the clavicle. Incidentally, it is the very first ossification center in the human skeleton. Shortly thereafter, consecutive ossification centers appear in the humeral, ulnar and radial shafts, followed by that in the scapula in week 8 of gestation.

The ossification process continues in two ways because at first the primary ossification centers appear in the shafts of long bones, followed by secondary ossification centers in their epiphyses and metaphyses [19]. With the use of ultrasound, primary ossification centers may be visualized in the first trimester of pregnancy, between weeks 7 and 12, whereas secondary ossification centers may be identified in the second and third trimesters of pregnancy [16].

In routine fetal ultrasound examinations, the length of the femur is critical in both determining fetal anatomy and assessing fetal ages. It is noteworthy that the evaluation of the length of long bones is extremely useful in the early detection of developmental defects [16]. If any skeletal dysplasia is suspected, it is necessary to measure the length of other long bones, including those of the upper limb: the humerus, radius and ulna [7].

According to Lee et al. [16], developmental defects of fetal bones can be separated into two main groups: generalized aplasias of the musculoskeletal system that influence the development of the entire fetus, and deformities that refer to selected parts of the fetus.

Having reviewed the professional literature about the ossification of the upper limb bones, we found those of the clavicle [1], humerus [19] and ulna [20] to be precisely quantified and expressed by growth curves. However, we failed to find any mathematical models concerning the primary ossification center of the radial shaft. This study is continuous with our research on the development of the upper limb bones.

Therefore, in the present study we aimed:

- to complete morphometric analysis of the radial shaft ossification center with reference to its linear, planar and volumetric parameters in the human fetus, so as to determine their normative age-specific values;
- to examine possible differences between sexes and sides for all analyzed parameters;
- to compute development dynamics for the analyzed parameters, expressed by best-matched mathematical models.

## Materials and methods

The study material comprised 47 human fetuses (25 males and 22 females) at the age of 17–30 weeks of gestation, originating from either spontaneous miscarriages or preterm deliveries. The material was acquired before the year 2000 and remains a part of the specimen collection at the Department of Normal Anatomy of our university. The experiment was sanctioned by the University Bioethics Committee (KB 275/2011). The fetal ages were determined on the basis of the crown–rump length. Table 1 lists the characteristics of the study group, including fetal age, number and sex.

**Table 1:** Age, number and sex of the fetuses studied

Gestational age (weeks)	Crown-rump length (mm)				Number of fetuses	Sex	
	Mean	SD	Min.	Max.		♂	♀
17	116.00	1.41	115.00	117.00	2	1	1
18	130.00	0.00	130.00	130.00	2	1	1
19	150.00	3.03	146.00	154.00	6	3	3
20	159.50	0.71	159.00	160.00	2	1	1
21	174.75	2.87	171.00	178.00	4	3	1
22	184.67	1.53	183.00	186.00	3	1	2
23	197.75	2.99	195.00	202.00	4	3	1
24	208.57	3.74	204.00	213.00	7	4	3
25	214.50	0.71	214.00	215.00	2	1	1
26	226.00	1.41	225.00	227.00	2	1	1
27	237.75	2.75	235.00	241.00	4	3	1
28	246.67	4.93	241.00	250.00	3	1	2
29	254.00	1.41	253.00	255.00	2	1	1
30	263.25	1.26	262.00	265.00	4	1	3
Total					47	25	22

**Fig. 1:**
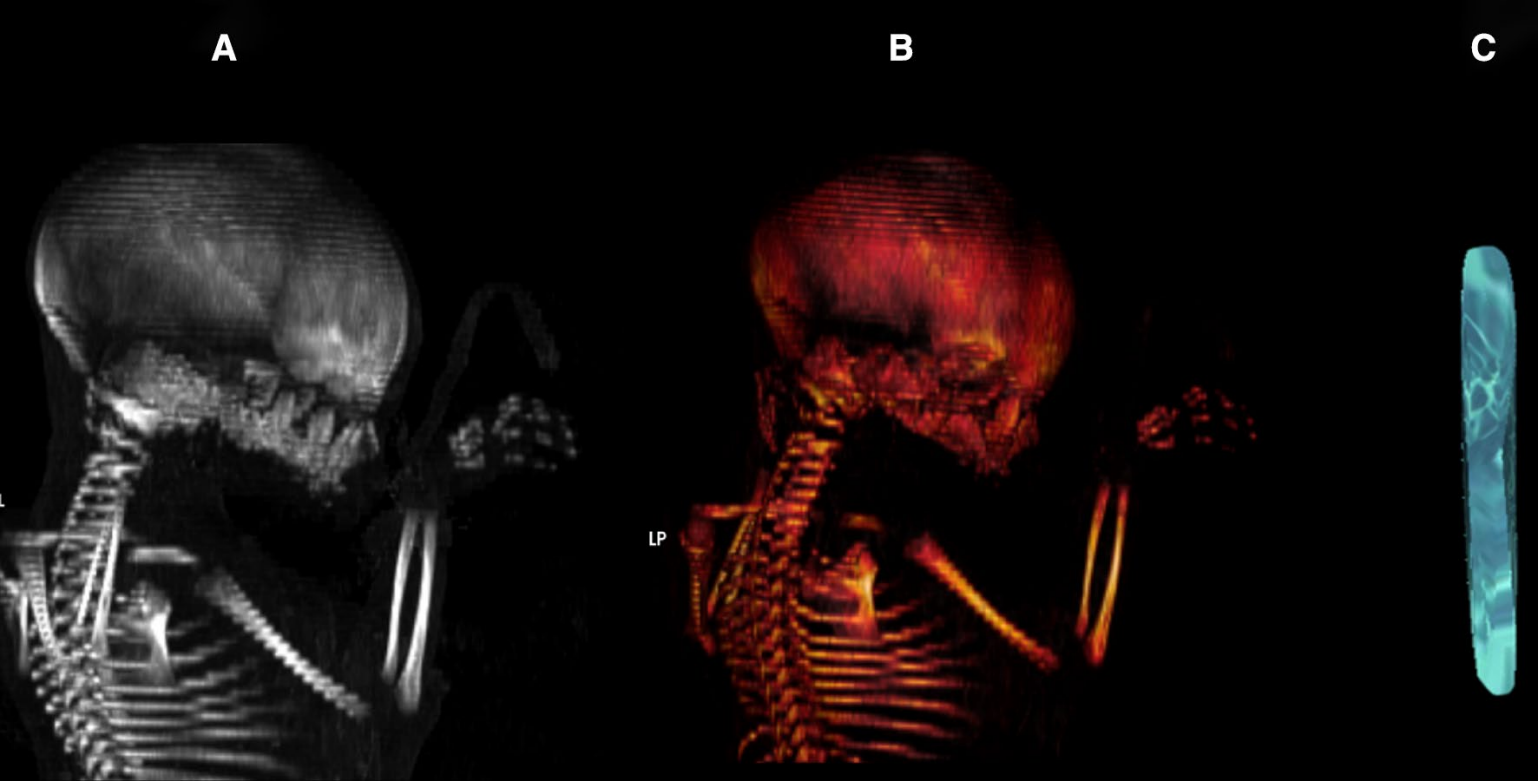
A male human fetus aged 26 weeks in the MPR projection with slice thickness 6.8 mm (**a**), its skeletal VRT reconstruction (**b**), and the volume ROI of radial shaft ossification center (**c**) using Osirix 3.9

With the use of a Siemens-Biograph 128 mCT camera (Siemens Healthcare GmbH, Erlangen, Germany) placed at Department of Positron Emission Tomography and Molecular Imaging (Oncology Center, Collegium Medicum of the Nicolaus Copernicus University, Bydgoszcz, Poland), all fetuses were scanned at a step of 0.4 mm, recorded in DICOM formats (Fig. 1), and successively subjected to morphometric analysis using the Osirix 3.9 software. Delineations of the radius ossification center were evidently visible [6, 10], thus enabling us to perform morphometric analysis in terms of its linear, planar and spatial parameters. Technical parameters of achieved CT images were as follows: gray scale ranged from − 275 to − 134 HU for a minimum, and from + 1165 to + 1558 HU for a maximum, window width alternated from 1.404 to 1.692, window level varied from + 463 to + 712, mAs = 60, kV = 80, pitch = 0.35, FoV = 180, rotation time = 0.5 s, slice thickness = 0.4 mm, image increment = 0.6 mm, and kernel = B45 f-medium.

This was a prerequisite to perform measurements of the primary ossification center of the radial shaft radial shaft, including its linear dimensions, projection surface area and volume, as follows:

1. length (in mm), based on the determined distance between the proximal and distal borderlines of the ossification center in the sagittal plane (Fig. 2);

**Fig. 2:**
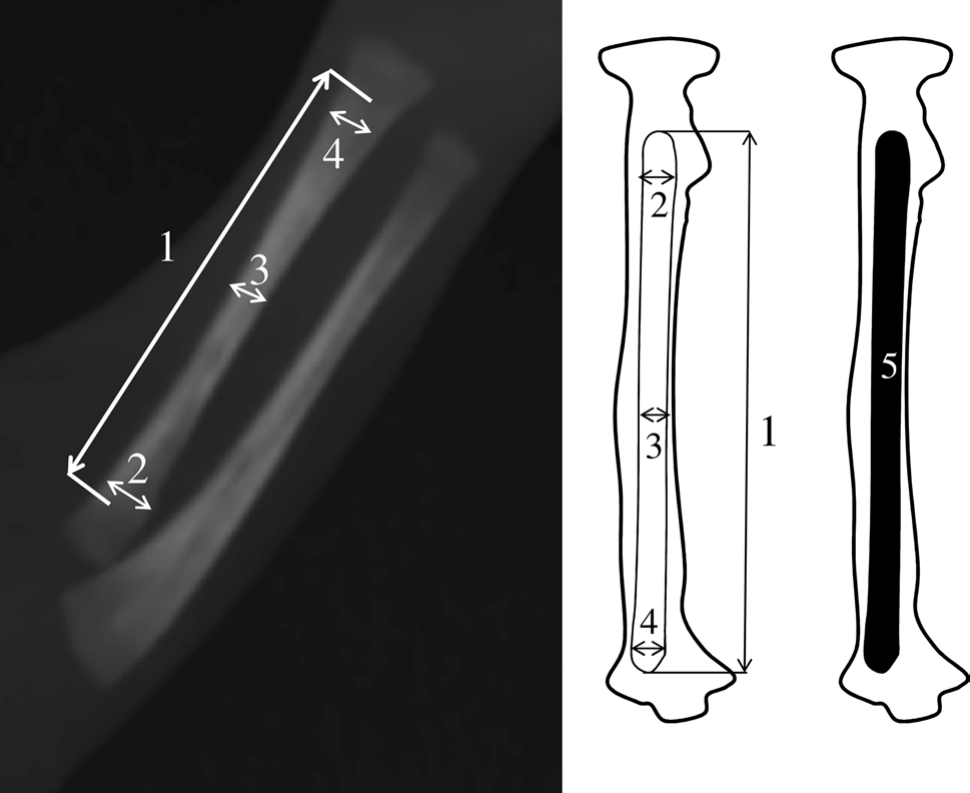
MPR projection and measurement scheme of the radial ossification center in the lateral view: 1—length, 2—proximal transverse diameter, 3—middle transverse diameter, 4—distal transverse diameter, 5—projection surface area
2. proximal transverse diameter (in mm), based on the determined distance between the medial and lateral borderlines of the proximal part of the ossification center in the sagittal plane (Fig. 2);
3. middle transverse diameter (in mm), based on the determined distance between the medial and lateral borderlines of the middle part of the ossification center in the sagittal plane (Fig. 2);
4. distal transverse diameter (in mm), based on the determined distance between the medial and lateral borderlines of the distal part of the ossification center in the sagittal plane (Fig. 2);
5. projection surface area (in mm^2^), based on the determined contour of the ossification center in the sagittal plane (Fig. 2), and
6. volume (in mm^3^), calculated using advanced diagnostic imaging tools for 3D reconstructions, taking into account the position and attenuation of radiation by bone tissues (Fig. 1c).

To precisely visualize and measure the primary ossification center radial shaft, the resulting fetal scans must have been rotated with relation to the three reference axes: vertical (cranial–caudal), horizontal and sagittal, to finally reach the reference position. It is noteworthy that in such a required position, the vertical, horizontal and sagittal axes always traversed the very center of the radial primary ossification center, and were set at the right angle to each other. Due to these maintained landmarks, the consistency in measurements was absolute. Additionally, such a position of these three axes made the radial primary ossification center to be set accurately in the frontal projection.

Our results were statistically analyzed. Distribution of variables was checked using the Shapiro–Wilk test, whereas homogeneity of variance was examined using Fisher’s test. The results were expressed as arithmetic means with standard deviations (SD). To compare the means, Student’s *t* test for independent variables and one-way analysis of variance with post hoc Tukey’s test were used. If no similarity of variance occurred, the non-parametric Kruskal–Wallis test was utilized. The characterization of developmental dynamics of all the analyzed parameters was expressed by linear and curvilinear regression analysis. The match between the estimated curves and measurement results was assessed using coefficients of determination (*R*^2^).

## Results

The mean values and standard deviations of all analyzed parameters of the left and right primary ossification centers of the radial shaft in human fetuses at the analyzed gestational ages have been presented in Tables 2 and 3 for length and proximal, middle and distal transverse diameters, and in Table 4 for projection surface area and volume.

**Table 2:** Length and transverse diameters for: proximal end, middle part and distal end of the right radial shaft ossification center in human fetuses

Gestational age (weeks)	*N*	length (mm)		Transverse diameter (mm)					
				Proximal end		Middle part		Distal end	
		Mean	SD	Mean	SD	Mean	SD	Mean	SD
17	3	16.7	0.2	1.3	0.1	1.2	0.1	1.2	–
18	3	16.9	–	1.8	0.2	1.3	–	1.3	–
19	5	17.5	0.4	2.3	0.1	1.4	–	1.4	–
20	3	18.7	–	2.5	–	1.5	–	1.6	0.1
21	4	21.2	1.6	2.6	0.1	1.5	–	1.9	0.1
22	2	24.6	0.6	2.8	–	1.7	–	2.2	0.1
23	3	25.9	0.7	3.1	–	1.8	–	2.4	0.1
24	6	28.0	0.8	3.6	0.1	2.0	0.1	2.5	–
25	3	30.0	0.4	3.8	–	2.1	–	2.6	–
26	3	31.6	0.6	3.9	–	2.2	–	3.0	–
27	5	32.5	0.1	4.2	0.2	2.3	0.1	2.9	0.4
28	2	32.8	0.1	4.5	–	2.4	–	3.3	–
29	2	33.5	–	4.7	0.1	2.4	–	3.4	–
30	4	35.3	1.5	5.0	0.2	2.7	0.2	3.8	0.2

**Table 3:** Length and transverse diameters for: proximal end, middle part and distal end of the left radial shaft ossification center in human fetuses

Gestational age (weeks)	*N*	Length (mm)		Transverse diameter (mm)					
				Proximal end		Middle part		Distal end	
		Mean	SD	Mean	SD	Mean	SD	Mean	SD
17	3	16.3	0.1	1.4	0.1	1.2	0.1	1.2	–
18	3	16.4	0.1	1.6	0.3	1.3	–	1.3	–
19	5	17.2	0.5	2.0	0.1	1.3	–	1.4	0.1
20	3	18.6	1.1	2.2	–	1.4	–	1.6	–
21	4	21.4	1.2	2.5	0.1	1.6	0.1	1.7	0.1
22	2	24.6	–	2.8	0.1	1.7	–	2.1	0.2
23	3	25.1	0.6	3.2	0.1	1.8	–	2.3	–
24	6	26.3	0.3	3.6	0.1	1.9	0.1	2.4	–
25	3	27.5	0.8	3.8	0.1	2.0	–	2.7	0.1
26	3	29.2	0.2	3.9	–	2.1	–	2.9	–
27	5	30.3	0.6	4.1	0.1	2.2	–	3.0	0.1
28	2	31.5	0.4	4.3	0.1	2.3	–	3.3	–
29	2	32.1	0.4	4.6	0.1	2.4	–	3.4	0.2
30	4	34.8	1.2	4.9	0.2	2.5	0.1	3.9	0.2

**Table 4:** Projection surface area and volume of the radial shaft ossification center

Gestational age	Number of fetuses	Projection surface area (mm^2^)				volume (mm^3^)			
		Right		Left		Right		Left	
		Mean	SD	Mean	SD	Mean	SD	Mean	SD
17	2	19.8	3.2	17.4	2.9	106.4	13.3	103.5	13.2
18	2	22.8	0.3	22.3	1.2	119.4	1.6	125.8	5.4
19	6	29.9	2.4	29.1	4.5	148.8	10.2	156.6	20.6
20	2	34.5	0.5	34.5	0.6	169.2	1.2	182.6	1.9
21	4	56.2	14.0	48.3	10.2	261.1	59.2	246.4	46.6
22	3	75.2	0.2	72.5	5.9	341.8	0.5	357.6	27.3
23	4	77.0	2.3	78.2	1.6	351.0	8.1	383.7	7.3
24	7	96.1	3.7	92.6	5.6	425.6	15.5	447.5	25.7
25	2	103.4	0.4	103.3	1.5	462.2	4.9	496.4	7.0
26	2	109.6	2.7	108.9	2.7	483.6	4.0	520.5	10.4
27	4	125.2	6.3	122.7	7.8	544.4	29.7	579.3	35.0
28	3	148.8	0.5	140.8	5.2	624.0	20.5	662.4	25.5
29	2	154.2	0.1	149.2	3.1	663.6	1.5	702.0	13.9
30	4	166.6	9.8	164.7	8.2	722.1	40.6	772.2	37.3

Since the statistical analysis revealed neither significant sex nor laterality differences, we computed one growth curve for each analyzed parameter. On both the left and right sides, the growth dynamics of the length and three transverse diameters of the radial shaft ossification centers followed linear functions.

**Fig. 3:**
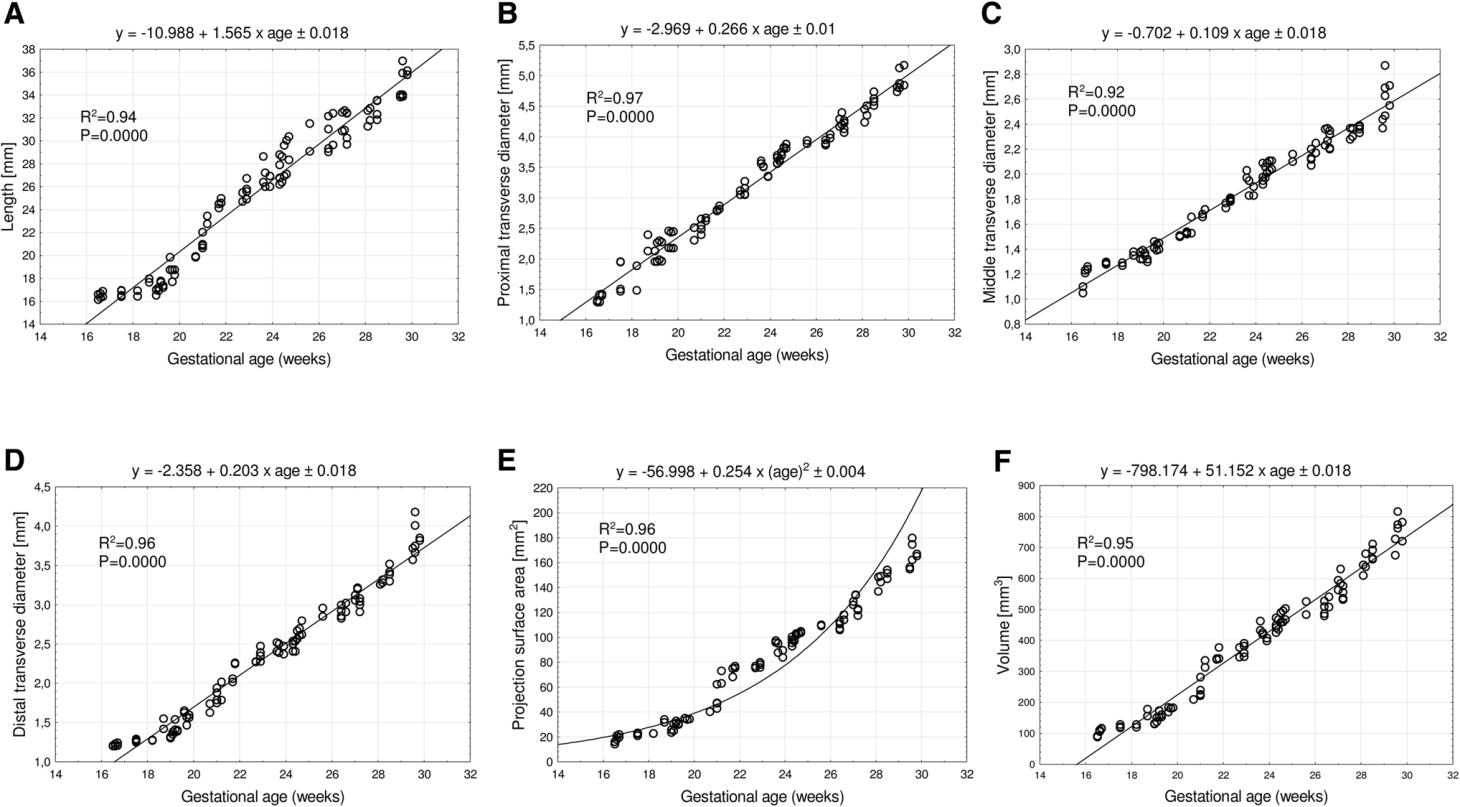
Regression lines for length (**a**), proximal (**b**), middle (**c**) and distal (**d**) transverse diameters, projection surface area (**e**), and volume (**f**) of the radial shaft ossification center

The mean length of the radial shaft ossification center at the range of 17–30 weeks increased from 16.7 ± 0.16 to 35.3 ± 1.5 mm on the right side, and from 16.2 ± 0.1 to 34.8 ± 1.2 mm on the left side, resulting in the linear function *y* = − 10.988 + 1.565 × age ± 0.018 (*R*^2^ = 0.94)—(Fig. 3a).

The mean proximal transverse diameter of the radial shaft ossification center ranged from 1.3 ± 0.1 mm at gestational week 17 to 5.0 ± 0.2 mm at gestational week 30 on the right side, and correspondingly from 1.4 ± 0.06 to 4.9 ± 0.17 mm on the left side, computing the linear function *y* = − 2.969 + 0.266 × age ± 0.01 (*R*^2^ = 0.97)—(Fig. 3b). The mean middle transverse diameter of the radial shaft ossification center at weeks 17–30 ranged from 1.2 ± 0.1 to 2.7 ± 0.2 mm on the right side, and from 1.2 ± 0.1 to 2.5 ± 0.1 mm on the left side, following the linear function: *y* = − 0.702 + 0.109 × age ± 0.018 (*R*^2^ = 0.92)—(Fig. 3c). During the study period, the mean distal transverse diameter of the radial shaft ossification center ranged from 1.2 to 3.8 ± 0.2 mm on the right side, and from 1.2 to 3.9 ± 0.2 mm on the left side, in correspondence with the linear function: *y* = − 2.358 + 0.203 × age ± 0.018 (*R*^2^ = 0.96)—(Fig. 3d).

Using our previous numerical data regarding the length of the ulnar shaft ossification center [20], the ulna-to-radius length index was calculated as a quotient of the length of ossification centers of the ulna and radius. The mean ulna-to-radius length index in the analyzed study period attained the value of 0.9 ± 0.1.

The mean projection surface area of the radial shaft ossification center ranged from 19.8 ± 3.2 mm^2^ at gestational week 17 to 166.6 ± 9.8 mm^2^ at gestational week 30 on the right side, and from 17.4 ± 2.9 to 164.7 ± 8.2 mm^2^, respectively, on the left side, following the quadratic function: *y* = − 189.992 + 11.788 × (age)^2^ ± 0.018 (*R*^2^ = 0.95)—(Fig. 3e).

Throughout the study period, the mean volume of the radial shaft ossification center revealed an increase from 106.4 ± 13.3 to 722.1 ± 40.6 mm^3^ on the right side, and from 103.5 ± 13.2 to 772.2 ± 37.3 mm^3^ on the left side. As a result, its volumetric growth followed the linear function: *y* = − 798.174 + 51.152 × age ± 0.018 (*R*^2^ = 0.95)—(Fig. 3f).

## Discussion

Knowledge of reference ranges for fetal limb bone measurements may support the diagnosis of skeletal dysplasias. However, it should be kept in mind that fetal biometry alone is not a sufficient tool [9]. Several studies have previously considered measurements and the degree of ossification of long bones in human fetal limbs [2, 5, 7, 12, 14, 22]. The appearance of ossification centers and their measurements may be considered real, because the skeleton maintains its integrity even in utero dead fetuses [2]. Measurements of both the total length and ossification center in long bones of the upper limbs (humerus, ulna, radius) might provide information that is useful in determining developmental age [2].

Brons et al. [5] calculated two indexes: the humerus-to-forearm bone length ratio and the ulna-to-radius length ratio, in 63 fetuses aged from 12 to 40 weeks of gestation. These two indexes for the 50th percentile were presented, as follows: 1.13 and 1.14 at week 12, 1.11 and 1.14 at week 16, 1.09 and 1.14 at week 20, 1.06 and 1.14 at week 24, 1.04 and 1.14 at week 28, 1.02 and 1.13 at week 32, 1.00 and 1.13 at week 36, and 0.98 and 1.13 at week 40, respectively. Zorzoli et al. [22] calculated the ulna-to-radius length ratio, which reached the value of 0.99 ± 0.12. It should be emphasized that in our study, the ulna-to-radius length ratio attained the value of 0.91 ± 0.03 for the analyzed period.

In an ultrasound study of 663 fetuses aged from 12 to 42 weeks of fetal life by Chitty and Altman [7], the growth of the radius length followed the function: *y* = 7983/(age)^2^ − 1698.6/age + 91.634 (SD = 0.046386 × age + 1.1933). Using ultrasound, Exacoustos et al. [12] measured lengths of long bones, including the radius in 2186 fetuses aged from 13 to 40 weeks of gestation. The mean radius length for the 50th percentile increased in accordance with the function: *y* = − 29.090 + 3.371 × age − 0.031(age)^2^.

Bareggi et al. [2] suggested that the evaluation of total length of bones may provide only debatable information relevant to the assessment of fetal age, whereas the ossification center may be considered an important parameter related to the degree of skeletal ossification.

With the use of X-rays, Khan and Faruqi [14] measured shafts of long bones in 34 autopsied fetuses from India that had been immersed in 10% formalin solution. The mean increase in radius length ranged from 4.5 mm in lunar month 3 to 53.50 mm in lunar month 10 of gestation. The greatest monthly increase in length of the radial shaft was 13.41 mm in month 5, with the mean radius length of 23.66 mm. The authors observed the length of the radial shaft to commensurately increase with fetal age. Using ultrasound, Bareggi et al. [2] measured the radius length in 58 autopsied individuals aged from 8 to 14 weeks of prenatal life. The authors measured the overall radius length and the length of its ossified parts. The overall length of the radius in fetuses with a CRL of 38–116 mm increased from 4.6 to 30.8 mm. Of note, the radius was longer on the right side in 20 fetuses, and on the left side in 3 fetuses. In turn, in fetuses with a CRL of 38–116 mm, the length of the ossified part of radius ranged from 1.8 to 22.4 mm.

This paper is the first report to morphometrically analyze the radial shaft ossification center in human fetuses with mathematical models of its growth dynamics. It is noteworthy to emphasize that the examined ossification center demonstrated no differences with respect to sex and laterality. Such a finding closely corresponded with the results of Baumgart et al. [1] concerning the primary ossification center of the clavicle, and those of Wiśniewski et al. [19, 20] regarding the ossification centers of the humerus and ulna in human fetuses. The present study emphasized that in fetuses aged 17–30 weeks, the radial shaft ossification center increased proportionately to fetal age, following the functions: *y* = − 10.988 + 1.565 × age ± 0.018 for its length, *y* = − 2.969 + 0.266 × age ± 0.01 for its proximal transverse diameter, *y* = − 0.702 + 0.109 × age ± 0.018 for its middle transverse diameter, *y* = − 2.358 + 0.203 × age ± 0.018 for its distal transverse diameter, and *y* = − 798.174 + 51.152 × age ± 0.018 for its volume. On the other hand, the projection surface area of the radial shaft ossification center increased following the quadratic function of fetal age in weeks: *y* = − 189.992 + 11.788 × (age)^2^ ± 0.018. It should be emphasized that the growth dynamics of the radial shaft ossification center were related to those of the ulnar shaft ossification center [20], which grew in a proportionate fashion to fetal age, as follows: *y* = − 8.476 + 1.561 × age ± 0.019 for length, *y* = − 2.961 + 0.278 × age ± 0.016 for proximal transverse diameter, *y* = − 0.587 + 0.107 × age ± 0.027 for middle transverse diameter, *y* = − 2.865 + 0.226 × age ± 0.295 for distal transverse diameter, *y* = − 821.707 + 52.578 × age ± 0.018 for volume. However, it is noteworthy that an increase in projection surface area of the ulnar shaft ossification center followed the quadratic function of fetal age in weeks: *y* = − 50.758 + 0.251 × (age)^2^ ± 0.016.

There are no reports in the professional literature about dimensions of the radial shaft ossification center, which indubitably precludes a more comprehensive discussion on this topic. However, the quantitative data of the radial shaft ossification center obtained in this study may play a crucial role in diagnosing skeletal dysplasias that are frequently characterized by disrupted or retarded growth of the antebrachial bones, e.g., rhizomelia, spondylodysplasia, achondrogenesis, thanatophoric dysplasia, TAR syndrome, VATER syndrome, Holt–Oram syndrome, Fanconi anemia and Edwards syndrome.

It is estimated that there are six limb abnormalities per 10,000 live births, with their incidence higher in the upper limbs compared to the lower limbs. The limb abnormalities occur more often unilateral, and are present more frequently on the right side [11]. Radius abnormalities can be isolated or associated with other malformations. Details of the fetal upper limb are often poorly delineated than those of the lower limb, partly because the fetus tends to move the upper limb more intensively than the lower limb. Inasmuch as flexed elbows may be difficult to visualize, extended elbows can be easily identified. At the elbow joint, the ulna starts more proximally than the radius, however, at the wrist the epiphyseal cartilages of the ulna and radius end at the same level [17].

Rhizomelia results in shortened proximal parts of limbs (humerus and femur), and also affects the antebrachial (radius and ulna) and crural (tibia and fibula) bones. Diaphyseal dysplasia affecting the shafts of long bones causes their enlargement, sclerotization, thickening of the cortical layer, thinning or enlargement of the medullary cavity. Malformations of long bones may also be accompanied by deformities of the spine, i.e., spondylodysplasia. Achondrogenesis and thanatophoric dysplasia are lethal, with a typical appearance of hypoplasia of long bones in the upper limbs, including the humerus [3, 4, 8, 21].

The measurements of the normal radial shaft ossification center may be of relevance in the diagnostics of congenital defects.

Aplasias of the radius are genetic congenital defects typical of TAR syndrome, VATER syndrome, Holt–Oram syndrome, Fanconi anemia and Edwards syndrome. TAR syndrome, also known as thrombocytopenia with radial aplasia or congenital hypoplastic anemia, presents a pattern of genetic congenital defects inherited in an autosomal recessive manner that are characterized by aplasia of the radius and hypomegakaryocytic thrombocytopenia [15]. Holt–Oram (Harris–Osborne) syndrome is a combination of genetic congenital defects caused by mutation of the *TBX5* gene encoding a transcription factor, which results in congenital heart defects and malformations of the upper limbs. Developmental disorders of the upper limbs include unilateral or bilateral hypoplasia or aplasia of the thumb, as well as hypoplasia or aplasia of the humerus or radius causing phocomelia [15]. Fanconi anemia is a genetic form of congenital aplastic anemia associated with bone malformations, including the upper limb bones, kidney and heart defects, as well as the predisposition to tumors. A literature review demonstrated that to date approximately 1200 individuals have been diagnosed worldwide [15]. Edwards syndrome is a combination of congenital defects caused by trisomy of chromosome 18, with its incidence estimated at 1 in 8000 births. At the early stages of embryonic development, the rate of occurrence of trisomy 18 is greater than at later periods of gestation. As many as 95% of fetuses with this defect are miscarried. Such a syndrome is characterized by numerous skeletal anomalies, including radial aplasias. Of note, as with Down’s syndrome, its incidence increases with maternal age. Besides, Edwards syndrome is four times more frequent in girls than in boys [15].

Employing X-rays to examine 379 autopsied fetuses aged 21–42 weeks, Pryse–Davies et al. [18] observed highly statistically significant sex differences, with the faster development of ossification centers in female fetuses. They also demonstrated the development of ossification centers to be either significantly retarded or accelerated in fetuses with lethal malformations. A clearly slower development of ossification centers was detected in fetuses with low birth weight associated with D-trisomy and E-trisomy, lethal dysplasia, as well as primary developmental defects of long bones. In turn, an accelerated development of ossification centers occurred in fetuses with anencephaly.

Routine ultrasound enables to diagnose developmental defects, such as skeletal dysplasias, based on reduced dimensions of long bones in relation to gestational age, conspicuous abnormal morphological features and bone mineralization, as well as the presence of fractures. However, the effectiveness of ultrasound examinations ranges only from 40 to 60%, therefore the use of ultrasound alone is not sufficient to make a comprehensive diagnosis. As a consequence, when any skeletal dysplasia is suspected, diagnostic imaging based on radiographic [18] and computed tomography [1] techniques is essential. To date, more than 200 skeletal dysplasias have been described, with their incidences ranging from 2.3 to 7.6 per 10,000 births [13].

## Conclusions

1. The morphometric characteristics of the radial shaft primary ossification center show neither sex nor bilateral differences.
2. The radial shaft primary ossification center grows proportionately in length, transverse dimensions and volume, and quadratically in projection surface area.
3. The obtained numerical findings of the radial shaft ossification center are considered age-specific reference of relevance in both the estimation of fetal ages and the diagnostics of congenital defects.

## References

[1] Baumgart M, Wiśniewski M, Grzonkowska M, Badura M, Dombek M, Małkowski B, Szpinda M (2016). Morphometric study of the two fused primary ossification centers of the clavicle in the human fetus. Surg Radiol Anat.

[2] Bareggi R, Grill V, Zweyer M, Sandrucci MA, Narducci P, Forabosco V (1994). The growth of long bones in human embryological and fetal upper limbs and its relationship to other developmental patterns. Anat Embryol.

[3] Bober MB, Taylor M, Heinle R, Mackenzie W (2012). Achondroplasia–hypochondroplasia complex and abnormal pulmonary anatomy. Am J Med Genet.

[4] Bonafe L, Cormier-Daire V, Hall C, Lachman R, Mortier G, Mundos S, Nishimura G, Sangiorgi L, Savarirayan R, Sillence D, Spranger J, Superti-Furga A, Warman M, Unger S (2015). Nosology and classification of genetic skeletal disorders: 2015 revision. Am J Med Genet A.

[5] Brons JTJ, van Geijn HP, Bezemer PD, Nauta JPJ, Arts NFTh (1990). The fetal skeleton: ultrasonographic evaluation of the normal growth. Eur J Obstet Gynecol Reprod Biol.

[6] Chano T, Matsumoto K, Ishizawa M, Morimoto S, Hukuda S, Okabe H, Kato H, Fujino S (1996). Analysis of the presence of osteocalcin, S-100 protein, and proliferating cell nuclear antigen in cells of various types of osteosarcomas. Eur J Histochem.

[7] Chitty LS, Altman DG (2002). Charts of fetal size: limb bones. BJOG.

[8] Cho SY, Jin DK (2015). Guidelines for genetic skeletal dysplasias for pediatricians. Ann Pediatr Endocrinol Metab.

[9] De Biasio P, Prefumo F, Lantieri PB, Venturini PL (2002). Reference values for fetal limb biometry at 10–14 weeks of gestation. Ultrasound Obstet Gynecol.

[10] Duarte WR, Shibata T, Takenaga K, Takahashi E, Kubota K, Ohya K, Ishikawa I, Yamauchi M, Kasugai S (2003). S100A4: a novel negative regulator of mineralization and osteoblast differentiation. J Bone Miner Res.

[11] Ermito S, Dinatale A, Carrara S, Cavaliere A, Imbruglia L, Recupero S (2009). Prenatal diagnosis of limb abnormalities: role of fetal ultrasonography. J Prenat Med.

[12] Exacoustos C, Rosati P, Rizzo G, Arduini D (1991). Ultrasound measurement of fetal limb bones. Ultrasound Obstet Gynecol.

[13] Goncalves L, Jaenty P (1994). Fetal biometry of skeletal dysplasias: a multicentric study. Ultrasound Med.

[14] Khan Z, Farugi NA (2006). Determination of gestational age of human foetuses from diaphyseal lengths of long bones—a radiological study. J Anat Soc India.

[15] Klopocki E, Schulze H, Strauss G, Ott CE, Hall J, Trotier F, Fleischhauer S, Greenhalgh L, Newbury-Ecob RA, Neumann LM, Habenicht R, König R, Seemanova E, Megarbane A, Ropers HH, Ullmann R, Horn D, Mundlos S (2007). Complex inheritance pattern resembling autosomal recessive inheritance involving a microdeletion in thrombocytopenia—absent radius syndrome. Am J Hum Genet.

[16] Lee S, Kim T, Lee H, Park J, Chung S, Jeon D (2013). Length measurement of fetal long bone and fetal anomaly detection. Webmed Central Obstet and Gynaecol.

[17] Mahony BS, Filly RA (1984). High-resolution sonographic assessment of the fetal extremities. J Ultrasound Med.

[18] Pryse-Davies J, Smitham JH, Napier KA (1974). Factors influencing development of secondary ossification centres in the fetus and newborn. A postmortem radiological study. Arch Dis Child.

[19] Wiśniewski M, Baumgart M, Grzonkowska M, Małkowski B, Wilińska-Jankowska A, Siedlecki Z, Szpinda M (2017). Ossification center of the humeral shaft in the human fetus: a CT, digital, and statistical study. Surg Radiol Anat.

[20] Wiśniewski M, Baumgart M, Grzonkowska M, Szpinda M, Pawlak-Osińska K (2018). Quantitative anatomy of the ulna’s shaft primary ossification center in the human fetus. Surg Radiol Anat.

[21] Zoetis T, Tassinari MS, Bagi C, Walthall K, Hurtt ME (2003). Species comparison of postnatal bone growth and development. Birth Defects Res B.

[22] Zorzoli A, Kustermann A, Caravelli E, Corso FE, Fogliani R, Aimi G, Nicolini U (1994). Measurements of fetal limb bones in early pregnancy. Ultrasound Obstet Gynecol.

